# A harmonised dataset for modelling select underutilised crops Across EU

**DOI:** 10.1016/j.dib.2023.109679

**Published:** 2023-10-13

**Authors:** Eranga M. Wimalasiri, Ebrahim Jahanshiri, Alessia Perego, Marco Botta, Sayed N. Azam-Ali

**Affiliations:** a Crops for the Future UK, National Institute of Agricultural Botany, 93 Lawrence Weaver Road, Cambridge CB3 0LG, UK; b Department of Export Agriculture, Faculty of Agricultural Sciences, Sabaragamuwa University of Sri Lanka, Belihuloya 70140, Sri Lanka; c Department of Agricultural and Environmental Sciences-Production, Landscape, Agroenergy, University of Milan, Via G. Celoria 2, 20133 Milan, Italy

**Keywords:** Crop diversification, Crop modelling, Neglected and underutilised crops, Sustainable agriculture

## Abstract

Whilst simulating crop performance in different environments can help fill the knowledge gap and improve the adoption of crops that are currently neglected and underutilised in conventional agrifood systems, lack of experimental data remains a barrier to widespread modelling of these crops. To date, no attempt has been made to collate sub-species crop data that are specifically suited for modelling underutilised crops. This article describes the first attempt to develop a database for crop modelling data with a focus on European underutilised crops. Following a pilot study to identify crops with the potential across the EU, a structured dataset of detailed experimental data was developed by analysing more than 500 agronomic studies that were published across European agroclimatic zones from 1972 to 2022. The dataset contains minimum information for calibrating basic crop models for any location in the EU provided that enough experimental and environmental data are available. More specifically, the database includes crop phenology, yield, management practices, geographic and pedo-climatic details of select underutilised and neglected species. The information underwent a curation procedure to ensure its quality. The collated database will be used in CropBASE, the global knowledge base for underutilised crops.

Specifications Table

**Table:** 

Subject	Agricultural and Biological Sciences (General)
Specific subject area	Agricultural diversification, neglected and underutilised crops, EU
Type of data	Table Chart Database
How the data were acquired	Datasets are a compilation of information that is collected from various sources including books, research articles, databases, website articles, experts and local communities growing underutilised crops, etc. The focus was on extracting data from literature sources that are peer reviewed, credible, and are primarily published in the English language. Link to the metadata of the studies. https://zenodo.org/record/8406516
Data format	Tabulated and linked data (secondary data), analysed, curated, further ingested into Global Knowledge Base for Underutilised crops
Parameters for data collection	The following are secondary data collated in the database: Taxonomy data: scientific name, variety or landrace Publication information: author, journal, year Geographic data: continent, country, site, latitude, longitude Soil data: Lower Depth, Clay (%), Sand (%), Silt (%), Texture, S.O., Bulk Density, Total carbon (%) Experimental data: Experiment duration (years), Seeding rate, Sowing depth, dates of each treatment /intervention. Production: yield, yield date, Above Ground Biomass (Agb), Agb date Phenology data: Growing Degree Days (sowing-to-harvest), emergence date, flowering date, maturity date Metadata (source of primary data): Study number, digital object identifier (DOI), web link to the source publication
Description of data collection	Credible sources containing experimental data, either from agronomy trials or meta-analysis containing experimental data were selected through literature search. As the focus of the work was to collate as much information as possible about agronomy trials of select underutilised crops, all data were collected and inserted into a shareable template on Google Docs. Units were harmonised to ensure their usability in crop modelling. The location of experiments were recorded in either address format (e.g. name of the center or location that the experiment is performed that can be reverse geo-located). All of these sources are linked with the dataset in the ‘Metadata’ sheet of the database (https://zenodo.org/record/8406516).
Data source location	Institution: Crops for the Future UK (CIC) City/Town/Region: Cambridge Country: UK All secondary information of primary data are described in the respected publication as recorded in the ‘metadata’ table of database with reference to the study that is cited
Data accessibility	Repository name: Zenodo Data identification number: 10.5281/zenodo.8406516 Direct URL to data: https://zenodo.org/record/8406516
Related research article	*E.M. Wimalasiri, E. Jahanshiri, A. Perego, S.N. Azam-Ali, A Novel Crop Shortlisting Method for Sustainable Agricultural Diversification across Italy, Agronomy. 12 (2022) 1636.*https://doi.org/10.3390/agronomy12071636

## 1 Value of the Data

• The dataset contains information about the basic crop growth (phenology, production, management practices, geographic and pedo-climate) of select underutilised and neglected species that were collected from published sources.
• Detailed and tabulated data are beneficial for variety of reasons, ease of use tabulation, search and filtering, ease of download and quality control, and ease of transfer and reusability.
• The data can be used by crop modellers to parameterise simple crop models provided initial evidence of productivity for selected underutilised crops. They can also be used for automated analysis procedures and mapping of yield across larger areas in the future.
• The lack of data for yield simulations using crop models is a barrier to crop diversification. The data can be used in crop models to evaluate the suitability and forecast the yield of underutilised crops.

## 2 Data Description

### 2.1 Basic details

**Fig. 1:**
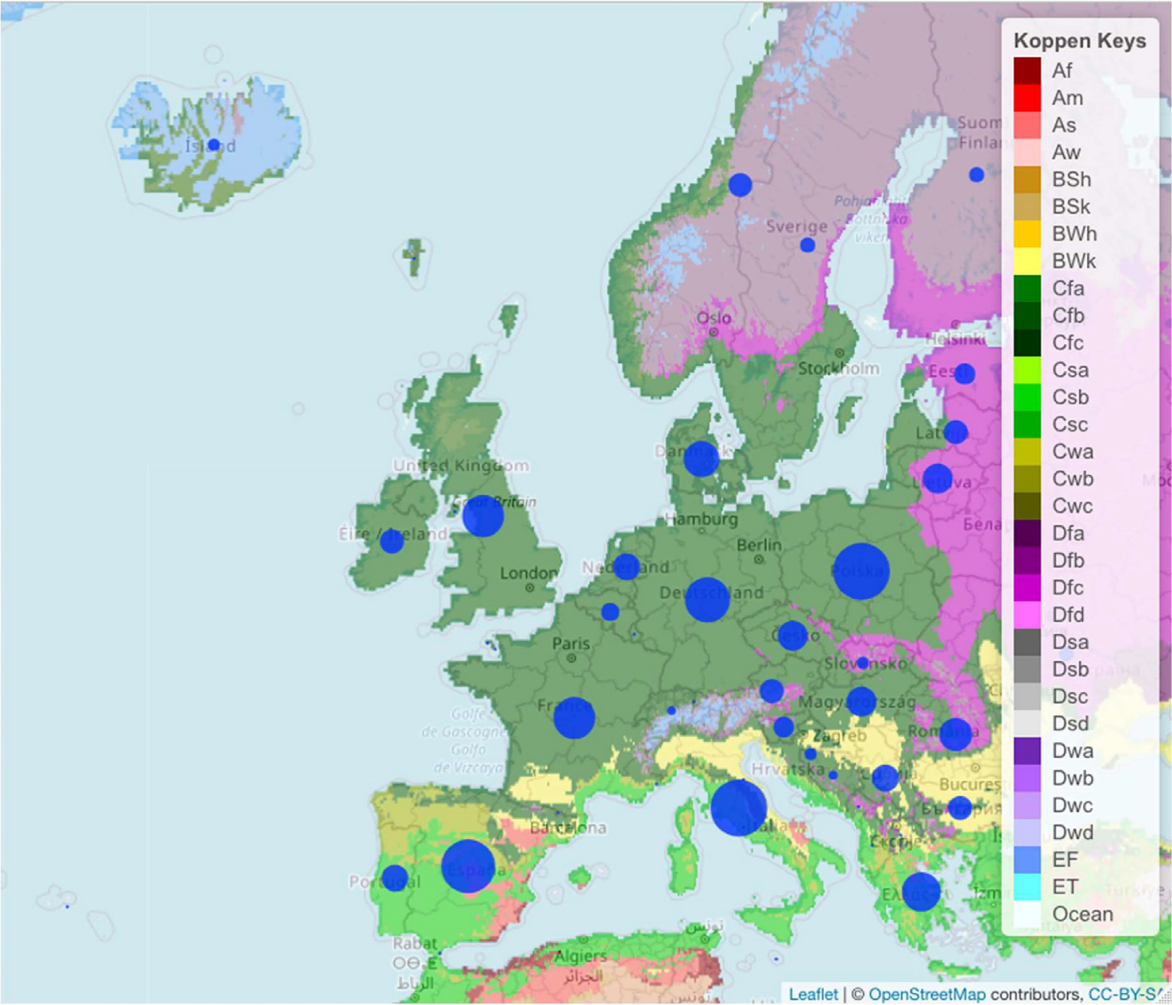
Distribution of underutilised crops across EU with added Köppen climate classification [1].

The database contains a total of 9,779 data points that were distributed among 35 countries in Europe (Fig. 1, Table 1). The highest and the lowest number of data points were collected from Italy (1,098) and the Republic of Belarus (1) respectively. More than 1,000 data points were collected from Poland (1,096). 100 data points were collected from 24 countries and less than 10 data points in 2 countries (Fig. 1).

**Table 1:** List of crops available in the database.

Scientific name		Common name	Comments
Ceratonia siliqua		Carob	Underutilised legume tree
Cichorium endivia		Endive	Underutilised vegetable
Eragrostis tef		Teff	Underutilised cereal
Ficus carica		Fig	Underutilised fruit tree
Helianthus tuberosus		Jerusalem artichoke	Underutilised starchy roots/tubers
Lentil Culinaris		Lentil	Pulse
Lupinus albus		White lupin	Underutilised legume
Malus domestica		Apple	Fruit
Malus pumila		Apple	Fruit
Medicago sativa		Alfalfa	Underutilised legume
Panicum miliaceum		Proso millet	Underutilised minor millet
Pisum sativum		Pea	Legume
Prunus avium		Cherry	Fruit
Prunus domestica		Plum	Fruit
Pyrus communis		Pear	Fruit
Rheum rhaponticum		Rhubarb	Underutilised vegetable
Setaria italic		Foxtail millet	Underutilised minor millet
Trifolium repens		Clover	Underutilised legume
Vicia faba		Faba bean	Underutilised legume
Vigna unguiculata		Cowpea	Underutilised legume

**Fig. 2:**
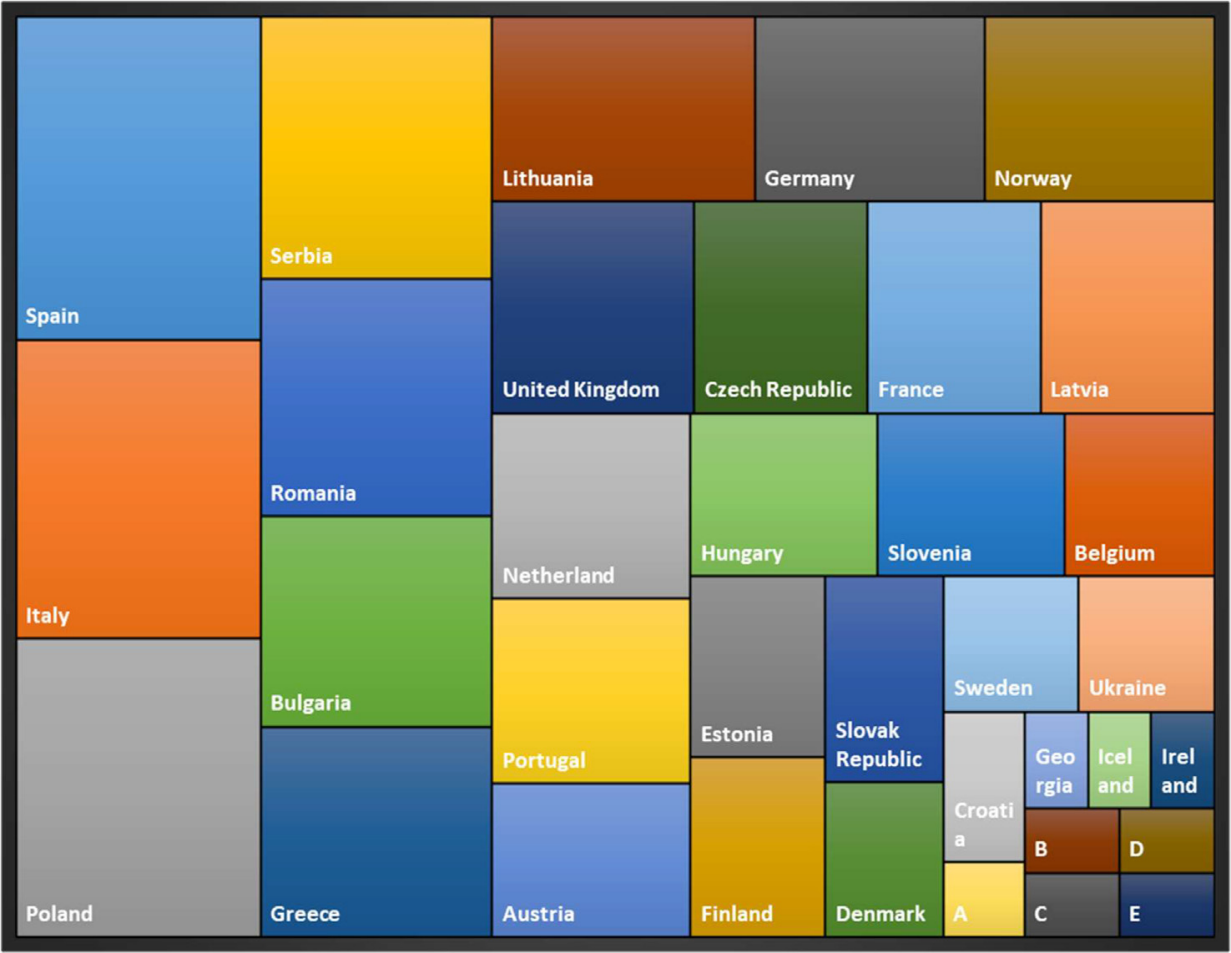
Comparison of the distribution of studies of underutilised crop species among countries, where A = Bosnia and Herzegovinia, B = Croatia, C = Republic of Belarus, D = Republic of Cyprus and E = Switzerland.

The collected data were distributed among 20 species (Table 1). No published information on field experiments was found in Europe for 13 crops that include *Chenopodium pallidicaule, Vigna subterranea L., Psophocarpus tetragonolobus, Hordeum vulgare, Phaseolus vulgaris, Triticum aestivum, Zea mays (underutilised cultivar), Solanum lycopersicum, Acacia victoriae, Peltandra virginica, Solenostemon rotundifolius, Cucurbita ficifolia* and *Nasturtium officinale*. Out of the 20 crop species, 5 or more than 5 crops were reported from 19 countries while 10 or more than 10 crop species were reported from 5 countries (Fig. 2). The highest number of crops (13 - *C. silique, C. endivia, H. tuberosus, L. Culinaris, L. albus, M. sativa, P. sativum, P. avium, P. domestica, P. communis, T. repens, V. faba* and *V. unguiculata*) were reported from Spain.

**Fig. 3:**
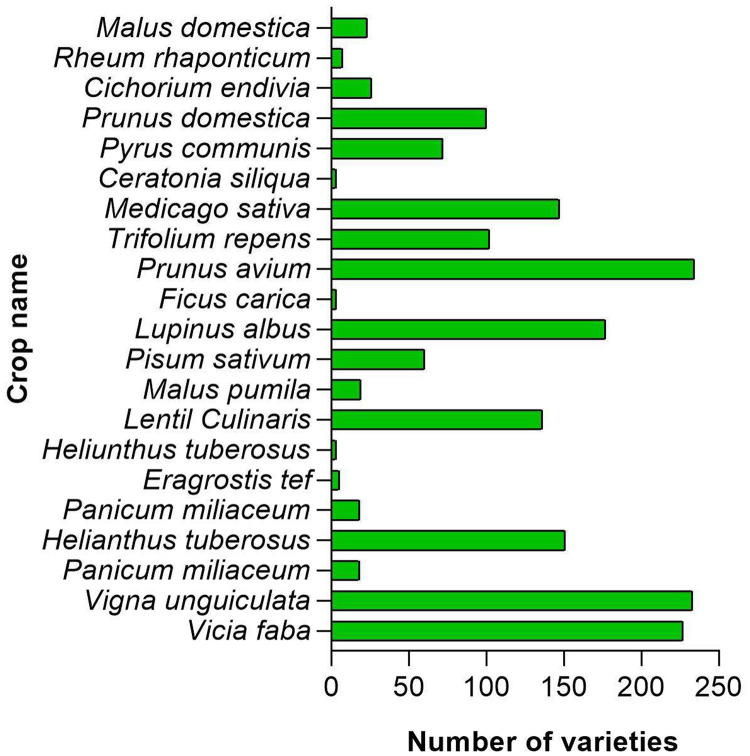
Distribution of varieties/landraces per crop.

Details on at least two cultivars/ varieties/ accessions were available for all the crops [2]. Altogether, details on 1,682 cultivars/ varieties/ accessions are available in the database where the highest number of cultivars are available on *Vigna unguiculata* (234) (Table 2, and Fig. 3). More than 100 were reported for 8 crops; *V. unguiculata, V. faba, L. albus, H. tuberosus, P. avium, M. sativa, L Culinaris* and *T. repens*.

**Table 2:** The cultivar details of the crops available in the database.

Scientific name	Common name
Ceratonia siliqua	Carob
Cichorium endivia	Endive
Eragrostis tef	Teff
Ficus carica	Fig
Helianthus tuberosus	Jerusalem artichoke
Lentil Culinaris	Lentil
Lupinus albus	White lupin
Malus domestica	Apple
Malus pumila	Apple
Medicago sativa	Alfa alfa
Panicum miliaceum	Proso millet
Pisum sativum	Pea
Prunus avium	Cherry
Prunus domestica	Plum
Pyrus communis	Pear
Rheum rhaponticum	Rhubarb
Setaria italica	Foxtail millet
Trifolium repens	Clover
Vicia faba	Faba bean
Vigna unguiculata	Cowpea

### 2.2 Publication information

The publication information of the collected data is important for scholars in two ways; (i) to gather more related information and (ii) to find a journal to publish similar information. Other than the name of the journal and the published year, the name of the first authors is also mentioned in the database which is helpful for research collaboration.

### 2.3 Geographic data

Other than the country, the name of the location/ experimental site is also available along with the latitude and longitude. Therefore, anyone who is interested in a specific location can obtain the data from the database. The duration of the individual experiments is also available to enable crop modeller to parameterise the models at different locations.

### 2.4 Pedo-climatic data

Different types of the available soil data include depth of the sample collection (m), clay (%), sand (%), silt (%), soil textural class, organic matter (%), bulk density (g cm^−3^) and total soil carbon (%). Due to the unavailability of the data in the publications, the climatic data for the experimental period and site is not available in the database.

### 2.5 Crop data

#### 2.5.1 Crop management

Different types of crop management data are available in the database; sowing rate, sowing depth, type, rate and date of fertiliser application, tillage, other soil treatments, type and rate and date of herbicide and pesticide applications.

#### 2.5.2 Phenology

Dates of emergence, flowering, maturity and other phenological details where applicable are available in the database. Also, the Growing Degree Days (GDD) to harvesting and other phenological stages are available.

#### 2.5.3 Production

Under the production category, the yield, above-ground ground biomass and the date of harvesting are available. The date of harvesting is available as the date, Julian date and the days after sowing/ planting.

### 2.6 Presentation of the data

Currently, the data are available in an online repository (https://zenodo.org/record/8406516). We are working on the migration of the data into Crops For the Future's Underutilised Crop Database (https://cropbase.co.uk/cropbasev5) [3,4].

## 3 Use-Cases

**Fig. 4:**
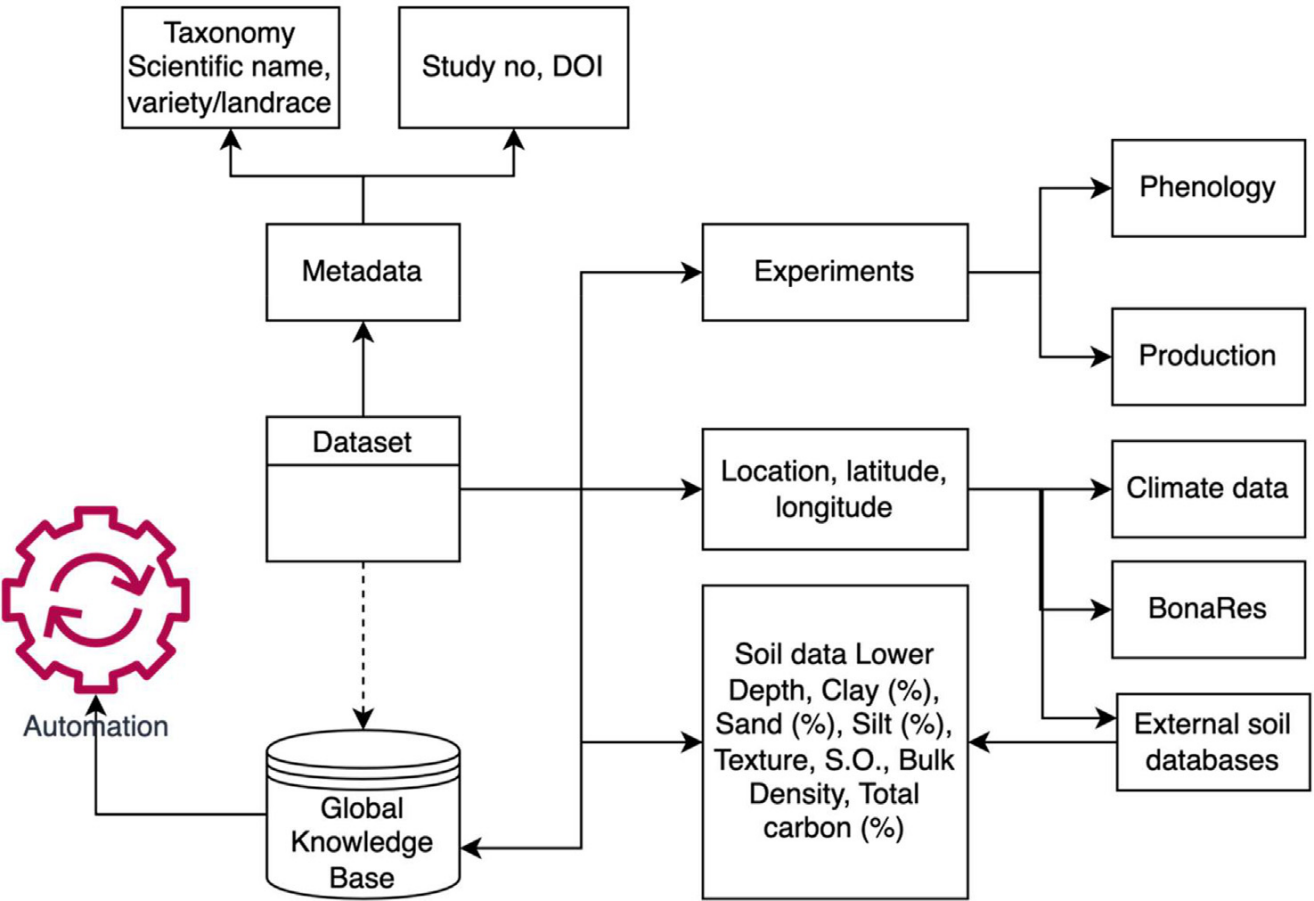
Future development of dataset

Digital crop databases for underutilised and neglected crops were previously published [3], [4], [5]. Even though these databases contain general information on nutrient values, ecology, yields and utilization status, they lack crop management and phenological data which hinders crop model applications. Fig. 4 shows a schematic for the next stage of development of the dataset. The data will be incorporated into the Global Knowledge Base for underutilised crops [3], and will be used to develop the underutilised crop tool in the LANDSUPPORT platform (www.landsupport.eu), which is an online decision support system developed for the EU region plus the UK [6]. In order to fill the gaps in environmental data, they can be obtained from European soil and climate databases (such as BonaRes https://www.bonares.de/research-data) and this will pave the way for yield simulation on other underutilised/ unknown crops to potentially diversify the cropping systems [7].

Different crop models require different types and amount of input data to parameterize. For example, some crop models such as Decision Support System for Agrotechnology Transfer (DSSAT) [8] requires relatively larger types of input data compared to ARMOSA [9] and SIMPLE [10]. The data available in the database can be used to parameterize any type of crop model since it contains different types of data. The ARMOSA model has been parameterized for faba bean (*Vicia faba* L.), one of the underutilised crops, using the dataset described here (*Wimalasiri et al, in prep*). The dataset will be used to parameterize other underutilised crop and other crop models with different parameter, file format and data structure requirements.

## 4 Experimental Design, Materials and Methods

The data collection and data entry process started in December 2021 and ended on 31^st^ April 2022. A database of published peer reviewed articles was acquired through literature search on Google Scholar and Scopus. All combinations of <crop_name> and <EU_countries> were searched manually and the articles were downloaded. The next step was to analyse the articles and extract information as much as possible from the text, tables and figures. To ensure the applicability of data, special focus was given to collect information on collecting the coordinates of the experimental data. This will ensure the applicability of calibrated models for select underutilised crops. To ensure maximum data extraction, the graph digitizer tool (http://getdata-graph-digitizer.com) was used to extract experimental data from the graphs from within published articles. Further consultation with the experts were done during the collection process to ensure data that are collected contains unit and scale. Further, the database underwent a thorough check by an experienced data curators to ensure the quality of the collected data. All the issues with the data points were discussed and resolved following a bi-weekly discussion between data collectors, curators and scientists.

## Limitations

This dataset overcomes the first barrier in developing widespread crop modelling datasets for underutilised crops across EU. Although the authors made every effort to extract all the information related to the experiments, lack of proper standards for describing agronomy experiments is led to presence missing information in this dataset. This problem can be resolved when proper standards such as Data Standards for Agricultural Field Experiments and Production (ICASA) [11].

## Ethics Statements

Not applicable.

## CRediT authorship contribution statement

**Eranga M. Wimalasiri:** Conceptualization, Methodology, Formal analysis, Data curation, Writing – original draft, Visualization. **Ebrahim Jahanshiri:** Conceptualization, Methodology, Data curation, Writing – original draft, Visualization, Project administration, Funding acquisition. **Alessia Perego:** Writing – original draft, Writing – review & editing, Project administration. **Marco Botta:** Writing – original draft, Writing – review & editing, Project administration. **Sayed N. Azam-Ali:** Writing – review & editing.

## Data Availability

A Harmonised Dataset for Modelling Select Underutilised Crops Across EU (Original data) (Zenodo)
